# Aphasia in Some of Its Practical and Less Known Aspects

**Published:** 1899-05-06

**Authors:** 


### APHASIA IN SOME OF ITS PRACTICAL AND LESS KNOWN ASPECTS.

Dr. Conolly Norman, of Dublin, draws attention to certain features in connection with aphasia and allied conditions in the current number of the *Journal of Mental Science* (April, 1899). It is pointed out that as speech preceded writing by many generations and centuries in the evolution of the race, so the centres for speech-utterance in the brain are better organised and have attained a greater fulness of growth in man than the centre governing the movements of writing. It also appears that, either through the influence of racial habit or because the muscles of the speech organs are more flexible, there seems to be a special facility for their use in efforts of expression. This appears from the curious fact that deaf mutes who have been taught lip language use that mode of communicating with each other in preference to dactylology (dumb alphabet), though the latter has generally been learned earlier, and is probably less liable to erroneous interpretation. In that kind of cerebral affection known as asymbolia the recognition of the uses and purposes of objects is lost, while the sensional functions which may be brought to bear upon such objects seem individually impaired. A patient of this kind cannot deal with articles in daily use. Show him a cigar, a knife, and a matchbox, and he is unable to cut the cigar and light it. Heilbronner has described three such cases of asymbolia, and gives the post-mortem appearances in two cases, viz., circumscribed foci of softening in the tempero-occipital lobes. In short, a limited lesion of some parts of the brain is capable of producing a condition of most profound asymbolia, in which conduct might be reduced to the level of profound childish ignorance (agnosia). In an interesting case published by Pick the patient exhibited the following symptoms: Irregular contraction of both R. and L. fields of vision, and marked psychical blindness; slight psychical deafness, but no word-deafness; marked loss of the power of cognising objects by the combined action of taste and smell; and a slight R. hemiplegia with paresis of speech utterance. In this case the autopsy revealed circumscribed foci of softening in the posterior half of the fusiform and lingual convolution on the R. side; while the posterior third of the lingual lobule, and almost the entire cuneus on the L. side were softened. In this, as in other and similar cases, the patient exhibited some enfeeblement of mental function, so that he failed to try to correct one sense by another. In other words, the rousing of associated images, consequent upon presentative stimuli proceeding from an object, seemed in abeyance. Thus Rabus showed that a patient of his who suffered from psychical blindness, seldom or never attempted to obtain recognition of an object by touch as ordinary blind people constantly do. A case recorded by Graskey is referred to, where the patient could recognise objects but could not name them, whereas on hearing the name he could at once point to the object. This patient also showed a marked inability to retain for any time either auditory images (memories of sounds), visual images, or symbols of any kind. Thus, if he was shown singly and successively the letters of a word he could not tell at the end what the word was, for he had already forgotten the first letters, though he could read the word when it was presented to him in its entirety. Again, if an object shown to him was recognised, and was then put away for a few minutes, it was found that on asking him now to touch the object named he could not do so, as the association of name with object had lapsed from his memory—*i.e.*, the nexus in memory did not persist. For the same reason he could not group together images in any class. Further observations on this case were reported by Wolff. He noted a general weakness of association, which the patient was able to overcome by the direct contemplation of an object with regard to which he wanted to speak. He further helped himself by writing down the name of the object. Thus, if he was asked the colour of leaves he looked out of the window in order to behold a tree, and then was able to write down the word "green." Looking at sugar he could only indicate (in writing) its visible properties, but did not know its taste until he put it in his mouth. Dr. Norman also refers to the well-known defects of symbolism observed in general paralysis and in epilepsy after fits, where for example a patient asked to repeat a spoken word failed to do so unless the written word was also shown to him, or where simple numerals could not be repeated from hearing alone but had to be supplemented or reinforced by holding up the proper number of figures before the patient could utter the word. The patient had an idea of the congruity of auditory and visual impressions, for if one uttered the word "three" and held up four fingers, he shook his head and remained silent. It is pointed out that these defects of the general paralytic are due to or associated with the somewhat widely-spread, rather than distinctly focal lesions occurring in his brain. Similarly in senile dementia it appears that the very common loss of correct cognition among such dements is an analogous condition, and that the resulting condition, viz., a loss of recognition of objects and the development therewith of ideas that they are not at home, that they are in strange places, &c., depends on cerebral degenerative changes comparable to those of psychical blindness or of general paralysis. The form of aphasia which least interferes with the power of thought expression, and in which mental integrity is best preserved, is that in which a lesion exists which is strictly limited to Broca's centre, the glosso-kinæsthetic centre of Bastian. In lesions involving other parts of the cortex there may be, according to the situation of the lesion, more or less confusion of auditory word memories with wrong use of words, and similarly of usual word memories with corresponding faults in writing. The defect may in other cases amount to a total psychical blindness as shown by Bastian in his recent work on aphasia, and there may be present in addition, as complications or concomitants, such conditions—"asymbolia" or "agnosia"—as have been referred to above, the localisation of which, however, is as yet far from satisfactory, though the importance of such studies is great.

